# Promoting Global Cardiovascular Health to Advance the Sustainable Development Agenda

**DOI:** 10.1016/j.jacadv.2024.101388

**Published:** 2024-11-20

**Authors:** Shanthi Mendis, Ian Graham, Asmus Hammerich, Bente Mikkelsen, Maryam Kavousi, Ramesh Pathirana, Evgeny Zheleznyakov, Jagat Narula

**Affiliations:** a Geneva Learning Foundation, Geneva, Switzerland; b Trinity College Dublin, Dublin, Ireland; c Director, UHC/Noncommunicable Diseases & Mental Health, WHO Eastern Mediterranean Regional Office (EMRO), Nasr City, Cairo, Egypt; d UHC/Communicable and Noncommunicable Diseases, World Health Organization Headquarters, Geneva, Switzerland; e Department of Epidemiology, Erasmus MC University Medical Center, Rotterdam, the Netherlands; f Ministry of Health, Colombo, Sri Lanka; g World Health Organization Regional Office for Africa, World Health Organization, São Tomé and Príncipe, Africa; h University of Texas Health Sciences Center, Houston, Texas, USA

**Keywords:** cardiovascular health, cardiovascular disease, universal health coverage, primary health care, noncommunicable diseases, inequalities, social and commercial determinants of health

## Abstract

The burden of cardiovascular disease has declined in high-income countries in the past 3 decades but is growing in low- and middle-income countries due to epidemiological, demographic, and socioeconomic shifts. A range of cost-effective policies and interventions are available for advancing cardiovascular health (CVH) through primordial, primary, and secondary prevention. We showcase multifaceted challenges that stifle the global progress of CVH including shortcomings in financial protection, health systems, primary health care, national health policies, service coverage, and surveillance. We highlight the under-acknowledged global disparities in health expenditure and health workforce capacities. We emphasize the need of addressing social and commercial determinants of health and a more granular analysis of challenges to implement context-appropriate national CVH responses, particularly in low- and middle-income countries. Finally, we propose progressive realization of universal health coverage and national health policy reform as sustainable strategies for overcoming the barriers to achieve CVH in order to reduce premature mortality from noncommunicable diseases by one-third by 2030 (Sustainable Development Goal target 3.4).

## Highlights

• Serious inequalities exist in the occurrence of cardiovascular diseases, which are declining in high-income countries and rising in low- and middle-income countries.
• Control of hypertension, diabetes, hyperlipidemia, and obesity by pharmacological means alone is neither affordable nor sustainable for low- and middle-income countries.
• To achieve cardiovascular health primordial, primary and secondary prevention have to be implemented through reform of national health policies and progressive realization of universal health coverage.
• Affordable and sustainable policies and interventions for improving cardiovascular health exist and should be more widely implemented with a focus on equity.

The global noncommunicable diseases (NCDs) burden, including cardiovascular disease (CVD), has grown from 47% of disability-adjusted life years in 2000 to 63% of disability-adjusted life years in 2019.^1^ During the same period, the share of NCD deaths has grown to nearly three-quarters of all annual deaths. In 2021, 20.5 million people died from CVD.^2^ Excluding COVID-19 deaths, major CVDs (ischemic heart disease and stroke) were the leading causes of age-standardized deaths globally in 2021.^3^ Deaths due to CVD between the ages of 30 and 70—the most economically productive age span—have increased from 5.3 million in 2000 to 6.2 million in 2019.^4^ The probability of dying between the ages of 30 and 70 from any CVD, cancer, diabetes, or chronic respiratory disease is higher in low- and middle-income countries (LMICs) compared to high-income countries (HICs) (Table 1).^4^ Aging, globalization of marketing and trade, and the exposure to behavioral and environmental risk factors are driving the global burden of NCDs.^5^, ^6^, ^7^, ^8^ By 2048, total annual deaths are estimated to reach nearly 90 million of which 77 million will be NCD-related deaths—a nearly 90% increase in absolute numbers over 2019.^9^

**Table 1:** Probability of Dying Between the Ages of 30 and 70 From Any Cardiovascular Disease, Cancer, Diabetes, or Chronic Respiratory Disease^4^

World Bank Income Group	2000	2010	2015	2019
Low-income	28.3	25.7	24.5	23.9
Lower-middle-income	25.8	24.2	23.1	22.4
Upper-middle-income	24.3	20.2	18.2	17.4
High-income	16.4	13.2	12.4	11.3
Global (in %)	22.9	19.9	18.5	17.8

The objectives of this paper are to: 1) highlight the multifaceted challenges including global disparities in health expenditure and health workforce capacities that stifle the global progress of cardiovascular health (CVH); and 2) emphasize the need for granular analyses and context-specific responses to overcome challenges and advance global CVH to promote sustainable development.

## 2 decades of advocacy and action

The priority accorded to NCDs including CVD in the global health agenda and the political commitment of countries to address them have gathered momentum in the last 2 decades.^10^ Collective actions by an array of agencies, global reports, and high-level events have contributed to this positive shift. They include the high-level meetings of the United Nations General Assembly Special Sessions,^11^, ^12^, ^13^ leadership and technical guidance of the World Health Organization (WHO),^10^^,^^11^ work of the UN Interagency Task Force on NCDs,^14^ Ministerial conferences,^10^^,^^15^ World Health Reports,^16^ global strategies on behavioral risk factors,^17^^,^^18^ the legally binding global treaty on tobacco control,^19^ Global NCD reports,^5^^,^^20^ the UN’s Agenda 2030 and its Sustainable Development Goals (SDGs),^21^ the global NCD action plan 2013 to 2020 (GAP-NCD),^22^ and a mobilized civil society and academia.

Although bilateral, multilateral, and other global actors have invested in health in LMICs, there is inadequate prioritization of funding to NCD activities. In 2018 in low-income countries (LICs), of total spending on NCDs, 37% came from domestic public funds, and 15% from external aid. In middle-income countries (MICs), 59% came from domestic public funds, and 2% from aid.^23^ In 2019, bilateral donors and private philanthropies contributed 41% each to Development Assistance for Health committed to NCDs.^24^ Bilateral actors contributed 68% of Development Assistance for Health for other areas of health. The NCD allocation in the health funding portfolio of key bilateral donors was inadequate (United Kingdom 1.7%, United States 0.5%, Germany 1.4%, France 1.6%, Canada 1.6%).^24^ Some of them also failed to specifically prioritize NCDs in their International Development Strategies.

## Effective policy options and clinical interventions

In 2013, the World Health Assembly (WHA) endorsed the GAP-NCD 2013 to 2020, which was extended to 2030 by the 72nd WHA^10^ in 2019. It provides a roadmap and menu of policy options to be implemented collectively by all stakeholders, to attain 9 voluntary global targets^22^ (Table 2). To assist LMICs to attain these targets, the WHA also endorsed a core set of cost-effective and high-impact interventions known as the “best buys”^5^^,^^10^^,^^22^^,^^25^^,^^26^ (Table 3).

**Table 2:** 9 Voluntary Global NCD Targets to be Attained by 2030^22^^,^^25^

1	A one-third relative reduction in the overall mortality from cardiovascular diseases, cancer, diabetes, or chronic respiratory diseases.
2	A 20% relative reduction in the harmful use of alcohol
3	A 15% relative reduction in prevalence of insufficient physical activity
4	A 30% relative reduction in mean population intake of salt/sodium
5	A 30% relative reduction in prevalence of current tobacco use
6	A 25% relative reduction in the prevalence of raised blood pressure or to contain the prevalence of raised blood pressure
7	Halt the rise in diabetes and obesity
8	At least 50% of eligible people (aged 40 years and older with a 10-year cardiovascular risk ≥20%) including those with CVD to receive drug therapy and counseling (including glycemic control) to prevent heart attacks and strokes
9	An 80% availability of the affordable basic technologies and essential medicines, including generics, required to treat major NCD in both public and private facilities

Global targets 1, 2, and 3 have been updated to align with SDG and other targets.^10^

CVD = cardiovascular disease; NCD = noncommunicable disease.

**Table 3:** WHO Best Buys for Prevention and Control of Cardiovascular Diseases, Cost of Implementation, and Return on Investment (Table Does Not Include Interventions for Cancer)^22^^,^^26^

Intervention Area	Interventions	Cost per Capita	Return on Investment per USD 1
Reduce tobacco use	Taxation Packaging Advertising, promotion, and sponsorship Smoke-free public policies Health education	USD 0.04	7.43
Reduce harmful use of alcohol	Taxation Advertising Availability	USD 0.05	9.13
Reduce unhealthy diet	Reformulate food to reduce salt Supportive environment Health education Packaging (front-of-pack labeling)	USD 0.04	12.82
Reduce physical inactivity	Health education		2.80
Manage cardiovascular disease, hypertension, and diabetes	Counseling and drug therapy based on total cardiovascular risk (to control diabetes mellitus, hypertension, hypercholesterolemia, and secondary prevention of CVD)	USD 0.75	3.29

WHO = World Health Organization; other abbreviation as in Table 2.

There are 13 population-based best buy interventions to address tobacco use, unhealthy diet, physical inactivity, and harmful use of alcohol^5^^,^^10^ and one integrated cardiovascular intervention which can be sustainably implemented in primary health care (PHC) in LMICs. It consists of assessment and reduction of total cardiovascular risk using hypertension, diabetes, or smoking as entry points. Together they promote CVH through primordial, primary, and secondary prevention.^5^^,^^25^^,^^26^

The additional cost of implementing the best buys in LMICs is estimated to be USD 1.27 per person per year. Every dollar invested in them yields a return of at least USD 7 by 2030. Implementing the best buys has the potential to prevent 17 million heart attacks and strokes, reduce premature mortality by 15%, and save 8.2 million lives by 2030.^25^^,^^26^

Recently, GAP-NCD Appendix 3 was expanded by the addition of less cost-effective interventions which can be implemented in settings with better resources.^27^ The Disease Control Priorities^28^ and NCD Countdown 2030^29^ initiatives also present cost-effective pathways to address the NCD burden.

## Progress made by countries is insufficient

**Figure 1:**
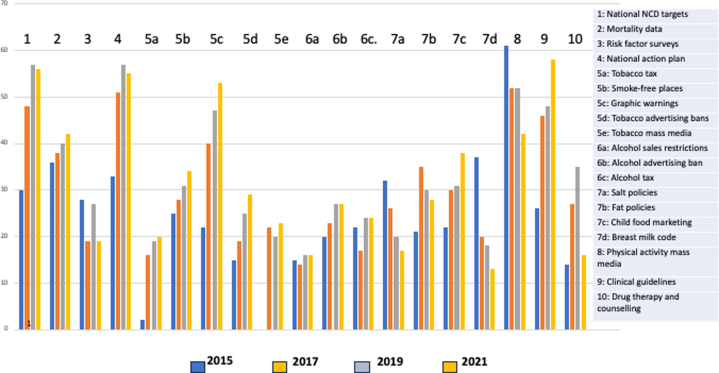
Percentage of Countries in Which Progress Indicators Are Fully Achieved: 2015, 2017, 2019, 2021^30^, ^31^, ^32^, ^33^

Data from WHO global country capacity surveys^30^, ^31^, ^32^, ^33^ show that from 2015 to 2021, the percentage of countries that had a national NCD action plan increased from 24% to 68%. Based on 14 progress indicators, the overall performance level between 2015 and 2021 has been modest (Figure 1). Only 4 indicators were fully achieved by more than half of countries by 2021. A statistically significant positive association is seen between performance on progress indicators and country income group for 58% of the indicators: mortality data, risk factor surveys, tobacco tax, tobacco and physical activity mass media campaigns, salt policies, fat policies, child food marketing, clinical guidelines, and drug treatment of those with high cardiovascular risk.^34^ Despite the low cost and good return on investment, implementation of best buys has been insufficient.

Although the prevalence of tobacco use is declining, the rate of decline is insufficient. Globally, total alcohol consumption per capita has declined since 2015 but consumption has increased in WHO’s South-East Asia and the Western Pacific regions. More than 1 in 4 adults and more than 80% of adolescents do not meet WHO’s recommended levels of physical activity. In 2016, more than 1.9 billion adults and 37 million children under 5 years of age were overweight. Globally, 99% of the population is exposed to air quality below the levels recommended by WHO. In 2021, 2.3 billion people were using polluting fuels and devices for cooking.^18^^,^^35^

Global, premature NCD mortality has declined from 22.9% in 2000 to 17.8% in 2019.^2^ However, the rate of change in most countries is too slow to achieve SDG target 3.4, which calls for reducing premature mortality from NCD by one-third by 2030.^2^^,^^21^ Based on 2010-2016 trends, women in 17 countries and men in 15 countries are expected to achieve SDG target 3.4.^36^ Although 56% of countries have set NCD targets, no country is on track to attain all of them by 2025.^18^^,^^35^^,^^37^

## Impediments to progress

Sustainable improvement of global CVH cannot be achieved through pharmacological or high-technology approaches alone. Social and commercial determinants play a critical role in shaping CVH. Policies that address these determinants need to be integrated into national health responses through multisectoral approaches. CVH also depends on aggressive primordial, primary, and secondary prevention of CVD^38^, ^39^, ^40^ implemented through national health policies and progressive realization of universal health coverage (UHC). Multifaceted challenges, which are most pronounced in LMICs, act as significant barriers to this process.^40^, ^41^, ^42^ Most of them are related to shortcomings in: 1) expenditure on health; 2) financial protection; 3) health workforce capacity; 4) service coverage; 5) health system and PHC; 6) national NCD policies; and 7) surveillance and health information systems. They are outlined below.

### Expenditure on health

**Figure 2:**
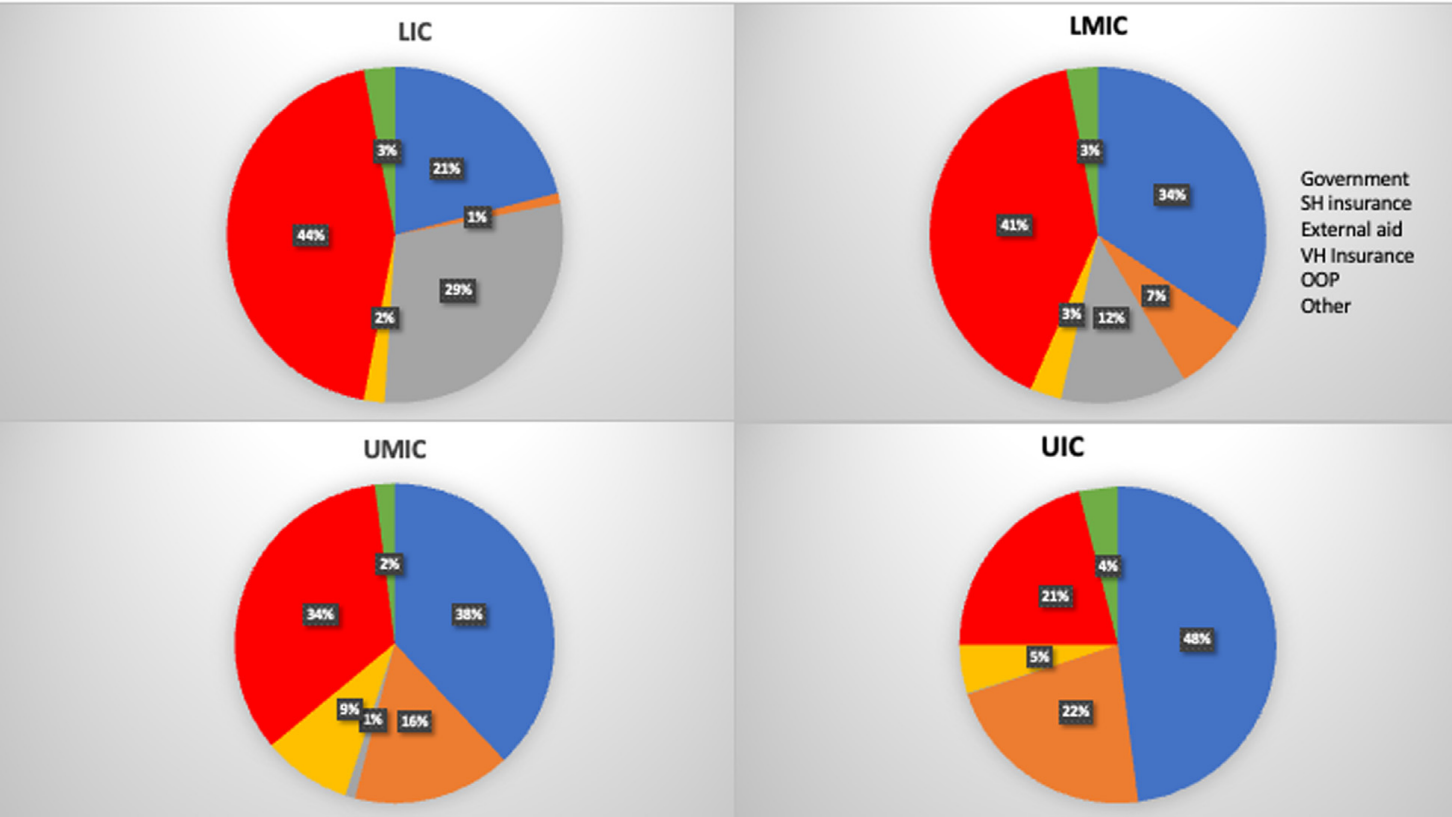
**Composition of Health Spending by Funding Source**^**43**^ Low-Income Countries (LICs), Lower-Middle-Income Countries (LMICs), and Upper-Middle-Income Countries (UMICs) Rely Heavily on OOP Spending. OOP = out of pocket; SH Insurance = social health insurance; VH Insurance = voluntary health insurance.

Global spending on health which has reached USD 9.8 trillion in 2021 is inequitably distributed.^42^ HICs (16% of the world population), account for about 79% of spending, upper MICs (UMICs) 17%, lower MICs 2.8%, and LICs only 0.24%.^43^ While government spending dominated in HICs (70%), health spending in LICs was mainly through out of pocket expenditure (OOPE) (44%) and external aid^44^ (Figure 2). In 2021, per capita health spending was USD 4001 in HICs, USD 531 in UMICs, USD 146 in lower MICs, and USD 45 in LICs.^42^ Across the 51 countries that reported health spending by diseases, an average of 26% of health spending went to NCDs. On average, 10% of external aid in these countries was allocated for NCDs. The largest share of spending on NCDs was from private sources (52%), followed by government sources (42%). Given the size of the burden, spending on NCDs was inadequate, accounting for about 30% of health spending in MICs and about 13% in LICs.^44^

### Financial protection

Global monitoring reports (2019 and 2021) on financial protection in health found an increasing share of the world’s population incurring catastrophic health spending.^45^^,^^46^ The majority of the 996 million people facing catastrophic health payments and the 505 million people pushed into poverty by OOPE was in LMICs.^45^^,^^46^ Early detection and long-term treatment are essential for preventing cardiovascular events such as heart attacks and strokes which can cause catastrophic health expenditure. However, many people (1.4-1.9 billion) delay seeking health services due to financial hardship.^44^^,^^45^ Furthermore, individuals with CVD are exposed to higher financial risks due to lifelong duration of care and unavailability of essential medicines in the public sector in LMICs. Although the latest WHO model list of essential medicines contains about 591 drugs,^47^ the majority of LMICs are not on track to provide even 80% access to the 11 essential NCD medicines (global NCD target 9). Moreover, there is a strong socioeconomic inequity in access to health services, with the disadvantaged being less likely to be diagnosed and effectively treated for NCDs.^48^

### Health workforce capacity

Lack of a skilled multidisciplinary health workforce is a major obstacle for promoting CVH in LMICs. Many cadres of health workers—public health professionals, PHC physicians, specialists, administrators, nurses, laboratory technicians, pharmacists, and community health workers—need to be available in adequate numbers to deliver a national NCD response. In addition, they also need to be competent, equitably distributed, empowered, and motivated to deliver quality care. Countries at all levels of socioeconomic development encounter difficulties in the training, retention, and performance of their health workforce.^49^^,^^50^ Currently, there is a severe shortage of health workers in LMICs which has been aggravated by the COVID-19 pandemic. Globally, the needs-based shortage of health care workers was estimated to be 17.4 million, of which 2.6 million are doctors and over 9 million are nurses^49^^,^^50^ (Table 4). The largest needs-based shortages of health workers are in South-East Asia at 6.9 million and Africa at 4.2 million. The global needs-based shortage of health care workers is projected to be still more than 14 million in 2030.^49^^,^^50^

**Table 4:** Stock of Health Workers in Millions 2013 (Global Health Observatory) and 2030 (Forecast)^49^

WHO region	Physicians		Nurses/Midwives		All Other Cadres		Total Health Workers		% Change
	2013	2030	2013	2030	2013	2030	2013	2030	
Africa	0.2	0.5	1.0	1.5	0.6	1.0	1.9	3.1	63%
Americas	2.0	2.4	4.7	8.2	2.6	3.4	9.4	14.0	50%
EM	0.8	1.3	1.3	1.8	1.0	2.2	3.1	5.3	72%
Europe	2.9	3.5	6.2	8.5	3.6	4.8	12.7	16.8	32%
SEA	1.1	1.9	2.9	5.2	2.2	3.7	6.2	10.9	75%
WP	2.7	4.2	4.6	7.0	3.0.	6.1	10.3	17.3	68%
Grand total	9.8	13.8	20.7	32.3	13.0	21.2	43.5	67.3	55%

EM = Eastern Mediterranean; SEA = South-East Asia; WP = Western Pacific; other abbreviation as in Table 3.

### Service coverage

The global progress toward UHC is monitored using the health service coverage index (SCI) and OOPE on health.^50^, ^51^, ^52^ The SCI (the average coverage of essential services based on 14 tracer indicators including NCD) improved globally from 45 to 68 out of 100 between 2000 and 2021.^52^ Across countries, SCI scores ranged from 28 to 91, with a strong positive association between SCI and countries’ income levels.^52^ The average SCI score observed in LIC was half that of the average score observed in HIC.^51^ While the service coverage of infectious disease, reproductive, maternal, newborn, and child health services have improved rapidly in the last 3 years, NCD services experienced much slower gains.^51^ One of the important reasons for this situation is the lack of expansion of health insurance to improve access to basic NCD services.^52^ NCDs need to be incorporated into the process of basic benefit package (BBP) design starting with the best buy interventions.^5^^,^^10^^,^^22^

### Health system and PHC

There are many health system shortcomings which need to be rectified.^53^, ^54^, ^55^, ^56^, ^57^, ^58^ Progress in PHC reform is hampered because of underfunding and allocation schemes that favor hospital care. For example, total expenditure on PHC in LICs and LMICs is USD 26 and USD 61 per capita, respectively. Government spending on PHC is USD 3 in LICs and USD 16 in lower MICs, which is much less than the WHO estimate of the per capita recurrent cost for PHC: USD65 in LICs and USD 59 in lower MICs.^44^^,^^55^ Other limitations include inadequately trained and remunerated workforce, difficulties in enforcing gate keeping arrangements leading to low utilization of PHC, inadequate public PHC facilities, lack of functional referral systems, weak community engagement, and failure in incorporating NCDs in BBPs.^56^, ^57^, ^58^ Tackling all these gaps are necessary for reform of PHC to advance CVH.

### National NCD policies

Policy reform to address social and commercial determinants of health that shape CVH is an inherently complex process. It involves engaging a range of stakeholders beyond the health sector, managing vested interests and maneuvering social, political, and cultural factors within the society.^59^, ^60^, ^61^ Capacity for policy analysis and reform is critical for implementation of population-level best buy interventions which address tobacco, alcohol, unhealthy diet, and physical activity (Table 3). Key barriers hindering NCD policy reform include resource constraints; legal loopholes; vested interests; low political priority; lack of local evidence to support policy implementation; insufficient skills of implementers; lack of environmental and systemic support;^59^, ^60^, ^61^ and inadequate funding and donor interest in NCDs.^62^

### Surveillance and health information systems

Weak surveillance and health information systems are major challenges. Many countries do not have a well-functioning civil registration and vital statistics system to register births and deaths. Nearly 40% of the world’s deaths are not registered, and cause of death is documented in only 8% of reported deaths in LICs.^63^ Risk factor surveys are not conducted regularly. In 2021, 82% of HICs, 70% of UMICs, 48% of lower MICs, and 35% of LICs had conducted a national adult risk factor survey.^34^ Only 55% of countries reported that patient records in PHC included NCDs. Nineteen percent were paper based, 41% were electronic, and 40% were a mix of the two. In the WHO’s African Region, only 4% of countries used an electronic health information system at PHC level.^34^ Most LMICs have limited capacity to collect good quality data and to utilize data to drive policy and planning.^35^

## Moving forward: sustainable strategies

First, the challenges alluded to above need to be addressed by contextualizing them to local circumstances. Second, severely limited workforce capacities and fiscal flows in LMICs call for a balance between the efforts to address NCDs and demands of responding to other competing health priorities. They include mental health, communicable diseases, maternal and child health, disease outbreaks, health consequences of aging, complex emergencies, and the climate crisis.^21^^,^^22^ Third, strategies adopted must have scalability and long-term financial sustainability. Achieving CVH is a political choice to tackle commercial interests and social and health disparities. It requires considerable strengthening of public health functions and health-system capacity through PHC, UHC, and cross-sectoral action.^64^ Adequate, predictable, and sustainable resources need to be mobilized through domestic, bilateral, and multilateral channels.^63^ Pro-UHC health financing policies must be implemented, raising revenue from government budgets and compulsory or voluntary prepaid insurance schemes and placing explicit limits on OOPE. Health taxes can also be used to minimize the adverse consequences of tobacco, alcohol, and unhealthy diet, while linking them to revenue generation for NCD activities.^65^^,^^66^

Highly impactful, cost-effective, and feasible interventions (NCD best buys) need to be prioritized in the national context and implemented to scale.^25^, ^26^, ^27^^,^^67^ Progress will depend primarily on an incremental increase of financial allocations for health and within that for NCDs. Higher investment in health is critical not only for equitable delivery of NCD services but also for scaling up public health functions of PHC and a broad range of health promotion activities: breast feeding, health in schools, workplaces, communities and cities, environment health promotion, and mass media campaigns.^68^ OOPE need to be reduced by incorporating NCDs in UHC financial protection schemes and BBP.^67^, ^68^, ^69^

Higher financial allocations are also necessary to strengthen PHC to ensure access to NCD services and equitable coverage.^68^^,^^69^ Basic diagnostics, technologies, and medicines, along with a competent workforce in sufficient numbers, must be made available for delivery of quality care. New technologies, including digital interventions, can be leveraged to scale up and support self-care and health literacy.^5^ Adopting an integrated PHC approach helps to reposition health systems to coordinate delivery of care across multiple NCDs.^5^ For example, at the first contact level, a total risk approach facilitates integration of programs for diabetes, hypertension, hyperlipidemia, and smoking cessation with other complementary programs. Such an approach avoids missing opportunities for prevention, diagnosis, and treatment, minimizes fragmentation of services and improves health system efficiency.^70^^,^^71^

In addition, policy coherence, cross-sectoral collaboration, multistakeholder engagement, and social mobilization are crucial for implementing marketing restrictions and legislative and regulatory measures to tackle social and commercial determinants and behavioral and environmental risk factors.^22^^,^^66^ Civil society needs to be encouraged to advocate for action and hold policymakers and government agencies to account. The private sector can be engaged for strengthening the national response taking due consideration of their potential conflict of interest with public health goals.^64^^,^^67^^,^^72^

Improving surveillance and monitoring (eg, periodic NCD risk factor surveys, country capacity assessments, health facility-level data) are essential in order to track progress and strengthen accountability.^5^^,^^22^^,^^63^^,^^67^ Finally, collaboration of international actors is needed to support national responses and facilitate translational research.^22^^,^^67^

The WHO’s GAP-NCD Road Map^67^ and an array of WHO action plans, tools, and technical packages^73^, ^74^, ^75^, ^76^, ^77^, ^78^, ^79^, ^80^ are available to provide guidance, facilitate adaptation and implementation of these strategies, and strengthen accountability. The Global Action Plan for Healthy Lives and Well-being for All^81^ brings together 13 multilateral health, development and humanitarian agencies to help countries to embed NCDs in the broader sustainable development agenda. Preparations have begun for the Fourth High-level Meeting of the UN General Assembly on NCDs which will be convened in 2025,^82^ including an international dialogue on sustainable financing for NCDs. They provide opportunities for all stakeholders to explore measures to accelerate implementation of national policies and plans, with financial and human resources allocated specifically to addressing NCDs.

## Conclusions

The CVH of individuals and populations is shaped by social and commercial determinants of health, behavioral, environmental, and cardiometabolic risk factors (tobacco, alcohol, unhealthy diet, physical inactivity, air pollution, obesity, diabetes, hypertension, and hyperlipidemia), aging, and other comorbidities (Central Illustration). To advance CVH, the focus has to shift from control of single risk factors in individuals to improving health outcomes of people and populations.^83^ Action on social and commercial determinants of health is essential throughout the life course, while strategically prioritizing high-impact affordable health services aimed at individuals and populations. In the face of multifaceted barriers and under-acknowledged large disparities in resources and capacities between countries, explicit embedding of NCD best buys in UHC represent a first step and a compelling approach to advance global CVH.

**Central Illustration:**
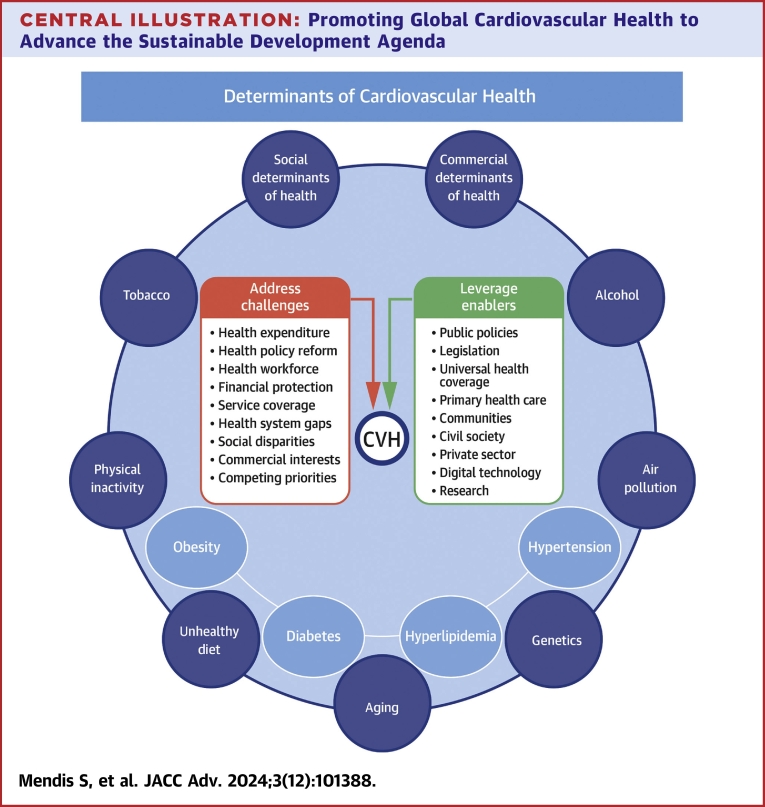
Promoting Global Cardiovascular Health to Advance the Sustainable Development Agenda CVH is determined by multifaceted factors. They include commercial and social determinants (poverty, education, urbanization, and food security), behavioral and environmental factors (tobacco, alcohol, unhealthy diet, physical inactivity, air pollution), cardiometabolic risk factors (obesity, hypertension, diabetes, and hyperlipidemia), aging, and genetics. Effective implementation of policies and interventions to achieve CVH requires overcoming challenges and leveraging enablers that influence them. CVH = cardiovascular health.

## Funding support and author disclosures

The authors have reported that they have no relationships relevant to the contents of this paper to disclose.
